# Status of household dietary diversity and associated factors among rural and urban households of Northern Uganda

**DOI:** 10.1186/s40795-023-00739-4

**Published:** 2023-07-10

**Authors:** Nelson Papi Kolliesuah, Solomon Olum, Duncan Ongeng

**Affiliations:** Department of Food Science and Post-harvest Technology, Gulu University, Gulu, Uganda

**Keywords:** Attitude, Dietary diversity, Gulu district, Knowledge, Rural, Urban

## Abstract

**Background:**

In Northern Uganda, 21 and 52.4% of children under five are underweight and stunted, respectively while 32.9% of pregnant women are anemic. This demographic situation suggests among other issues, a lack of dietary diversity among households. Good nutrition practices that confer dietary quality such as dietary diversity are known to depend on nutrition knowledge and attitude and are shaped by sociodemographic and cultural factors. However, there is a paucity of empirical evidence to support this assertion for the variably malnourished population of Northern Uganda.

**Methods:**

A cross-sectional nutrition survey was conducted among 364 household caregivers (182 from two locations in Northern Uganda; Gulu District (the rural) and Gulu City (the urban), selected through a multistage sampling approach. The aim was to determine the status of dietary diversity and its associated factors between rural and urban households of Northern Uganda. The household dietary diversity questionnaire and the food frequency questionnaire on a 7-day reference period were used to collect data on household dietary diversity whereas multiple choice questions and the five points Likert Scale were used to determine knowledge and attitude toward dietary diversity. Consumption of ≤ 5 food groups were regarded as low in dietary diversity, 6–8 food groups as medium and ≥ 9 as high dietary diversity score using the FAO 12 food groups. An Independent two-sample t-test was used to differentiate the status of dietary diversity between the urban and rural areas. The Pearson Chi-square Test was used to determine the status of knowledge and attitude while Poisson regression was used to predict dietary diversity based on caregivers’ nutritional knowledge and attitude and their associated factors.

**Results:**

The 7-day dietary recall period revealed that dietary diversity was 22% higher in urban (Gulu City) than in the rural area (Gulu District) with rural and urban households achieving medium (score of 8.76 ± 1.37) and high (score of 9.57 ± 1.44) dietary diversity status, respectively. Diets in both locations were dominated by starchy cereals and tubers while animal-source foods and fruits and vegetables were the least consumed. A higher proportion (51.65%) of urban respondents had good nutrition knowledge toward dietary diversity compared to their rural counterparts (23.08%) and a significantly higher proportion (87.91%) of the former exhibited positive attitude towards dietary diversity than the rural counterparts (72.53%). Application of the Poisson regression shows that nutritional knowledge was a positive predictor of dietary diversity in the rural (β = 0.114; *ρ* = 0.000) than in the urban areas (β = -0.008; *ρ* = 0.551). Caregivers attitude had no significant effect across locations. In terms of associated factors, marital status is a positive predictor of dietary diversity in the urban (β = 1.700; *ρ* = 0.001) than the other location (β = -2.541; *ρ* = 0.008). Whereas education level of household caregiver and household food expenditure show negative effects across the two locations, the educational level of the household head is an outlier as it positively predicted dietary diversity in the rural (β = 0.003; *ρ* = 0.002) when compared to urban area (β = -0.002; *ρ* = -0.011).

**Conclusion:**

Rural households in Northern Uganda have medium-level dietary diversity with urban households having high dietary diversity. Diets in both locations are dominated by starchy cereals and roots and tubers. The urban–rural food divide can be harmonized through nutrition education and outreach, specifically focusing on the FAO 12 food groups. Attitude toward consumption of fruits and vegetables which are seasonally abundant would improve dietary diversity and nutritional outcomes in the study area.

## Introduction

Globally, the prevalence of under-nutrition is on the rise. This is illustrated by the increase in the number of undernourished people from 615 million in 2017 to 768 million in 2020 [1]. A similar situation exists for overnutrition exemplified by the prevalence of overweight and obesity which increased from 22.1 and 8.7% to 25.8 and 13.1% [2], respectively over the last 18 years [3]. In Africa, the situation is more pressing because overweight and obesity coexist with under-nutrition [4]. Whereas food production is on the increase in several low-income countries in Africa including Uganda, food abundance without dietary diversity may not guarantee good nutrition outcomes [4]. Dietary diversity refers to the number of food groups consumed over a reference period and can be measured at household and individual levels [5]. If consumption of all food groups is achieved, then dietary diversity is considered high. However, if the consumption level is below three (3) and five (5) food groups per day and per week, respectively, dietary diversity is considered to be low. On the other hand, if consumption level falls in the range of four (4) to five (5) per day or six (6) to eight (8) food groups per week, then the diversity is considered to be medium. Nutritionally sound dietary diversity refers to consumption of six (6) or more food groups per day [6] or nine (9) or more food groups over a week [7]. Given the variation in nutrient composition of foods, consumption of diverse food groups is an indicator of good nutrition and nutrition-related health wellbeing.

The increase in food production observed in Africa over the last decade could be a pointer towards attaining good nutrition outcomes in the continent but this has not been the case especially with vulnerable groups of people (e.g., women, children, and the elderly) who continue to suffer from undernutrition challenges. Remarkable differences in food consumption behavior exist between rural and urban households in low-income countries. Rural diets are composed of indigenous food resources, obtained largely through own production pathway and are mostly dominated by starchy cereals and tubers [4]. This is not withstanding the fact that diverse foods are produced in the rural areas [8]. On the other hand, urban areas are usually flooded with variety of food products. Despite availability of diverse foods in the market, and the higher disposable income, urban people in low-income countries tend to buy few food groups or fast foods which are often limited in diversity [9]. Several studies exist on the differences in food production and access versus the amount of calories consumed between rural and urban households [4, 10, 11]. However, the underlying reasons for the disparity between rural and urban localities are largely unknown. As such, designing strategies to achieve adequate dietary diversity in low-income countries has been a major challenge because of limited information on the predictors of dietary diversity among households in such localities [12]. Previous studies [13, 14] indicated that nutritional knowledge, attitude and household socio-demographic characteristics are key factors that determine achievement of dietary diversity because they influence food consumption behavior. However, the effect of these variables on dietary diversity in rural and urban locations with varying abundance of food typical of low-income country settings is less understood.

Gulu City and Gulu District are areas in Acholi sub-region of Northern Uganda where under-nutrition indicators are high. The latest demographic health statistics indicate that 21 and 52.4% of children under five are underweight and stunted, respectively while 32.9% of pregnant women are anemic [15, 16]. This demographic situation suggests among other issues, lack of dietary diversity among households. Previous studies that examined consumption of fruits and vegetables in Acholi-Sub region showed disparity in consumption levels between rural and urban households [17]**.** This suggests that disparity in dietary diversity exists between rural and urban households. Whereas dietary diversity is known to depend on nutrition knowledge, attitude and sociodemographic factors [18], the information is largely derived from studies that focused on child and maternal nutrition [19] but little is known about how those factors influence dietary diversity at a household level which is reflective of the overall household nutrition well-being. In addition, limited information exists on how nutritional knowledge, attitude and sociodemographic characteristics influence household dietary diversity in the context of rural–urban divide typical of low-income country settings such as Northern Uganda. Therefore, using Gulu district and Gulu City as a case area, this study examined the status of dietary diversity and the influence of nutritional knowledge, attitude and sociodemographic factors on dietary diversity among rural and urban households in Northern Uganda.

## Methods

### Study area

**Fig. 1:**
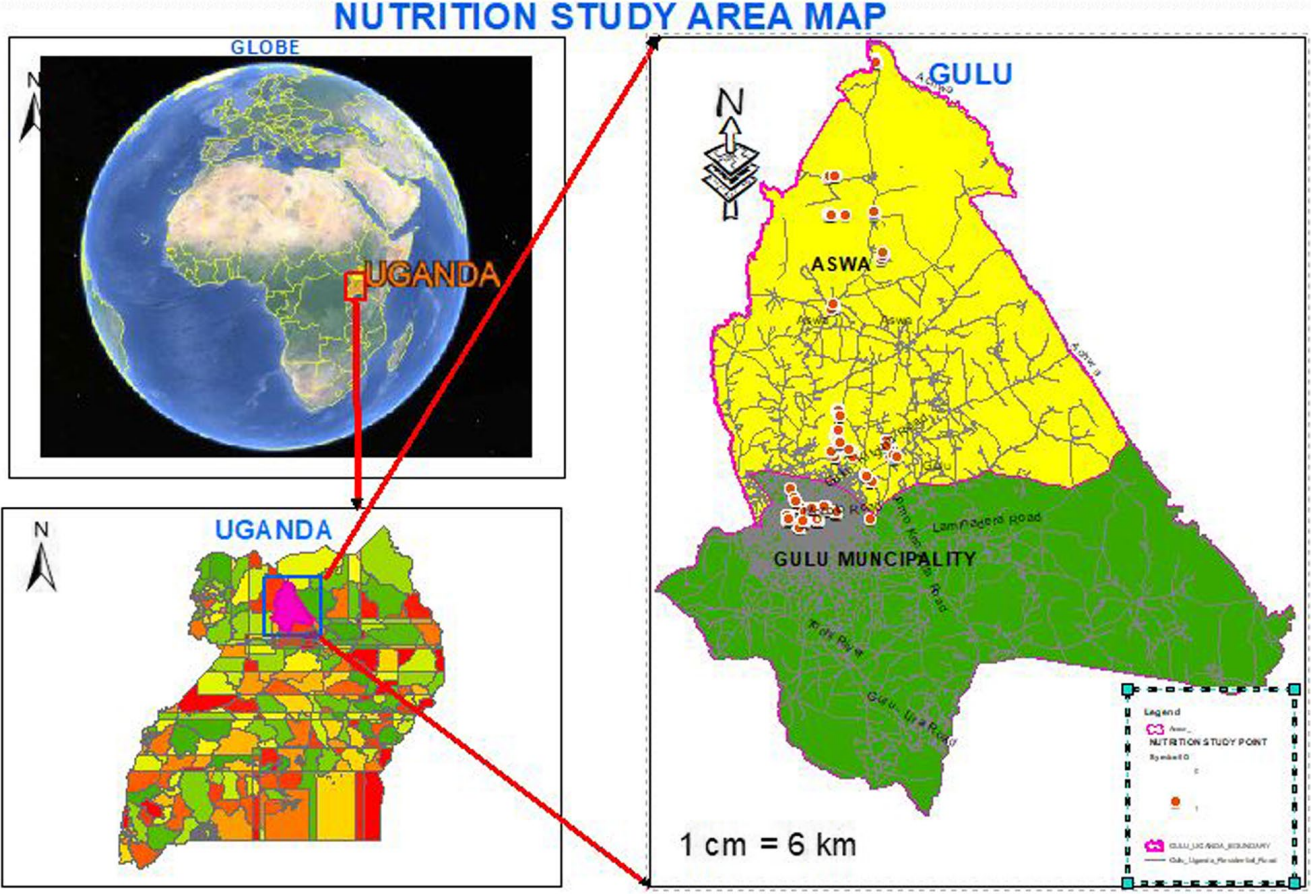
Map of Uganda showing the location of the study area (Gulu)

The study was conducted in Gulu City (the urban) and Gulu district (the rural), located in Northern Uganda. The area is situated at latitude 02°49′50.0"N, 32°19′13.0"E and longitude of 2.830556 and 32.320278, respectively. Gulu City and Gulu District have a combined household number of 55, 441 and an average household size of 5.0 [20]. Gulu City is divided into two divisions (BarDege-Layibi and Laroo-Pece) with a population of 150,306 which is 54.5% of the overall population of both the district and the City [20]. Gulu District is divided into six (6) sub-counties (Awach, Paicho, Bungatira, Palaro, Patiko and Unyama) with a total population of 125,307 (45.5%). The City and District are largely inhabited by the Acholi tribal community that speaks the Acholi language. Northern Uganda was chosen because of the high prevalence of undernutrition and over nutrition standing at 28 and 7.4%, respectively [21]. The map showing the study area is presented in Fig. 1.

### Study design, population and participants

A cross-sectional research design employing household survey was used to collect quantitative data from study participants. The choice of the design was deemed suitable because dietary diversity measurement is based on recall of food groups consumed over a 7-day or 24-h period and can be determined at one point in time. The study population consisted of households in Gulu district and City. The study participants were household caregivers, defined as persons who are responsible for food preparation in households. In low-income country context and the study area in particular, caregivers are usually women aged 18 – 59 years [22]. In addition, caregivers were used because they are always aware of all foods brought and prepared in households.

### Sample size and sampling framework

The required sample size (n) for the study was determined using a standard formula according to [10]. The formula is presented below:

1 $$n=\frac{\mathrm z^2\mathrm{pq}}{\mathrm e^2}$$

where n is the sample size, z is confidence level at 95% (standard value = 1.96), p is the proportion of malnourished people in Northern Uganda and was chosen to be 31.4% [23], e is the level of precision set at 5% and q is the proportion of the population that has good nutritional status (1-p) = (1–0.314 = 0.686).

2 $$\mathrm{Therefore}, n= \frac{{\left(1.96\right)}^{2}\left(0.314\right)\left(0.686\right)}{{0.05}^{2}}=331$$

However, considering a non-response rate of 10%, the sample size was adjusted upwards by the same percentage leading to a final sample size of 364. A multistage sampling procedure (four stages) was applied to select participating households and respondents. In the first stage, two (2) divisions and two (2) sub counties were randomly selected from the four (4) divisions in Gulu City and six (6) sub-counties in Gulu district, respectively. In the second stage two (2) parishes were randomly selected from each division (for Gulu City) and a Sub-county (for (Gulu district) while in the third stage three (3) villages were randomly selected from each of the parish selected in the second stage. This resulted into 12 villages each from Gulu district and 12 in Gulu City. Stage four, a systematic random sampling technique was used to select participating households. Households were listed in alphabetical order and assigned unique identification (ID) numbers. A list of households was generated through the local council leaders. Thereafter, fifteen (15) households per village were systematically selected by picking every third household on the list, resulting into 364 households (182 from rural and 182 from urban). One participant who was a caregiver from each selected household participated in the study.

### Data collection

#### Assessment of dietary diversity

Dietary diversity questionnaire (HDDQ) and the non-quantitative food frequency questionnaire (FFQ) were jointly used to collect data for measuring household dietary diversity. The HDDQ and FFQ were jointly used in order to provide a more elaborate understanding of the disparity between rural and urban households in terms of dietary diversity. The HDDQ utilized twelve (12) food groups which have been used in similar African setting [6]. These food groups include cereals, vegetables, fruits, meat, eggs, tubers and roots, fish and other seafood, legume nuts and seeds, milk and milk products, oil and fats, sweets and spices, and condiments and beverages. During data collection, participants were asked on whether or not certain food groups were consumed by their households in the last seven (7) days preceding the survey [24] to assess their level of varied diets on the food group consumed [25–27]. As applied in other studies [24], the 7-day reference period presents a better understanding of household nutrient adequacy which is a proxy measure of nutritional status. In the process of assessment, if a participant reported to have prepared mixed dishes, a full description of the ingredients was requested. For each food group, participant’s response was categorized and recorded as “yes or no”. A score of ‘1″ was given to a ‘yes’ response for each food group if the participant reported that the household consumed at least one food item from a particular food group during the past 7-day prior to the survey. Similarly, a score of ‘0’ was given to a ‘no’ response for a particular food group if the household did not consume any food items from that food group. The scores from the food groups were counted and used to create a household dietary diversity score (HDDS) for individual household according to the FAO guideline.

The procedures for administering the FFQ were adapted from [28–30] in which study participants responded to a list of food items on how often they consumed a given food (frequency) in the last 7-day preceding the survey. The frequency of each food consumed was assigned a code. A code of ‘0’ was assigned if the participant reported that the household “never,” consumed the food, ‘1’ for “once a day,” ‘2’ for “two or more times daily,” ‘3’ once per week, ‘4’ for “2–3 times per week”, ‘5’ for “four or more times weekly” according to [30]. The list of foods in the questionnaire was adapted and modified to include only foods relevant in Northern Uganda (Acholi sub-region in particular). Responses from the participants were used to generate food consumption frequency for each household ranging from a frequency of ‘1’ through 7.

#### Assessing status of knowledge and attitude toward dietary diversity

The study utilized a multiple-choice test comprising of ten (10) questions for measuring nutritional knowledge of participants. As applied in other studies [31], it is the best option for testing knowledge especially when all questions show the same number of options with only one of the answers being correct. In this case, each participant responded to questions relating to household dietary diversity. Knowledge response was awarded ‘0’ if the respondent chose either ‘wrong response or responded “don’t know’ whereas ‘1’ was awarded for ‘correct response’ as applied by [10, 32]. All questions were adapted from the general nutrition knowledge questionnaires for adults proposed by [33]. Only questions related to determining household dietary diversity were selected, modified and adapted to suit the scope of the study. Household nutritional attitude was assessed on what participants thought about dietary diversity [28]. Participant’s responses to the specific attitude statements used in the study were scored on a 5-points Likert scale. A score of ‘1’ was awarded if the participant ‘Strongly disagree’ to the statement, 2 for ‘disagree’, 3 ‘neutral, 4 ‘agree’ and 5 ‘strongly agree’.

### Data quality assurance

The study instruments (questionnaires) were pre-tested to check the appropriateness of the questions using test–retest reliability. This was done in Layibi and Pece divisions. Five percent (5%) of the actual sample size was used for the pilot process. The pre-tested areas were excluded from the list of places in which the sample was drawn. All steps of this pilot process were subjected to debriefing in order to finalize the tools for the next phase (actual data collection). The final version of the data collection instruments and the informed consent forms were translated into Acholi (the local language). High-quality checks that included enumerator’s audit and high-frequency checks were performed at all stages of the data collection exercise to ensure completeness and quality data. All households with incomplete questionnaires were revisited and interviewed accordingly.

### Statistical analysis

Data collected was entered, sorted, cleaned and coded into Microsoft Excel 2016 and exported to SPSS version 25.0 and Stata version 14.0 for analysis. In determining the status of nutritional knowledge toward dietary diversity, all correct responses from participants were given a value of ‘1’ and ‘0’ for incorrect and don’t know responses to create a score for each participant. Scores on all the specific nutritional knowledge questions were generated by summing the scores from each question and ranked as poor or good. In this case, knowledge was regarded as good when the score was > 50% [34] and the reverse for ​a​ poor​ response​. For attitude, participant’s responses to the specific attitude statements used in the study were used to create attitude score. Negatively worded statements were reversed during coding. Attitude was regarded as positive when the score was > 57% [34] and reverse if the score was less. Overall scores for knowledge and attitude were analyzed using descriptive analysis (frequencies and percentages) and summarized using graphs. The differentials in the status of knowledge and attitude between urban and rural households were determined using the Pearson Chi-square Test.

The Poisson regression as previously applied [35] was used to determine the knowledge, attitude and sociodemographic estimators of dietary diversity. The generalized linear function of the model, the derivative of its average [35, 36], and the final empirical function [37] used are presented in Eqs. 3, 4, and 5, respectively.

3 $$g\left[E\left(y\vert x_1,x_2,\dots,x_k\right)\right]=\beta_0+\beta^Tx_k;y\left|x_k\sim D\left(\theta\right),\right.$$

4 $$log\left[E\left(y\vert x_1,x_2,\dots,x_k\right)\right]=\beta_0+\beta_1x_1+\dots+\beta_kx_k;y\left|x_k\sim P\left(\theta\right)\right.$$

5 $$g\left(y\right)=log\left[E\left(y\left|x_k\right.\right)\right]=\beta_0+\beta_1x_1+\beta_2x_2\;\dots+\beta_kx_k+u_i,$$

Where: β and β_0_ represent coefficients of the vectors and the intersection term, respectively; g (·) is the link function of the linear model; D(θ) represents the exponential distribution at parameter θ [38], y is the household dietary diversity score, a count dependent variable; β_0_ is the intercept; β_1_, β_2_,... β_k_ are vectors of unknown parameters to be estimated; xk is a vector of explanatory variables i; u_i_ is a robust standard error term. Eight explanatory variables were used, details of which are covered in the result section. Stata version 14.0 was used to perform the analysis.

## Results

### Characteristics of households and participants surveyed

There were more male-headed households among both rural (68.7%) and urban (74.2) households surveyed (Table 1).

**Table 1:** Demographic characteristics of study participants (*n* = 364)

**Socio-demographic Variables**	**Location of residence**			
	**Rural**		**Urban**	
	**N**	**%**	**N**	**%**
Sex of Household Head				
Male	125	68.7	135	74.2
Female	57	31.3	47	28.5
Marital Status				
Single	14	7.7	16	8.8
Divorced	19	10.4	12	6.6
Married	22	67	133	73.1
Widowed	27	14.8	21	11.5
Education of Household Head				
No formal education	3	1.60	5	2.7
Primary	69	37.9	33	18.1
Secondary	44	24.2	64	35.2
Diploma/Certificate	8	4.4	25	13.7
Degree	4	2.2	13	7.1
Education of Caregiver				
No formal education	21	11.5	14	7.7
Primary	127	69.8	90	49.5
Secondary	27	14.8	59	32.4
College level education	6	3.3	18	9.8
Occupation of Household Head				
Not employed	5	3.9	3	2.1
Employed (Salaried)	17	13.4	51	36.4
Small scale trading	27	21.3	52	37.1
Causal Laborer	33	26.0	20	14.3
Farming	103	81.8	42	30.0
Retired Pension Earner	2	1.6	3	2.1
Occupation of Caregiver				
Not employed	6	3.3	36	19.8
Employed (Salaried)	5	2.7	22	12.1
Small scale trading	47	25.8	82	45.1
Causal Laborer	39	21.4	16	8.8
Farming	167	91.8	85	46.7
Retired Pension Earner	1	0.5	1	0.5
**Other Variables**				
**Continuous Variables**	**Mean**	**Standard Deviation**	**Mean**	**Standard Deviation**
Age of Caregiver	37	17	33	13
Household Monthly Income (UGX)	312,005	105,654	554,354	271,604
Household Monthly Food expenditure (UGX)	87,291	52,635	205,297	107,306
Household Size	5.80	2.87	5.43	2.61
Number of Income earner in household	1.74	0.61	1.68	0.57

The vast majority of these participants were married. The results further show that most respondents in the rural areas had completed primary education (69.8%), and were mainly engaged in farming compared to the urban area where majority had attained secondary education and were engaged in both farming and small-scale trade. A similar result was observed for household head in the rural area for which primary level of education was dominant while secondary education qualification was most pronounced among those in the urban area. Moreover, the average size of households across the two localities was five people with one to two people in each household earning some income. Food production and purchase were the most dominant means of obtaining food across the two locations.

### Level of consumption of different food groups

In Table 2, we present the mean frequencies of consumption of each of the twelve food groups captured across rural and urban households over a 7-day period. The results show that cereals were consumed more frequently than other staple foods, with urban households consuming them about 4.12 times weekly compared to rural households (3.65 ± 1.65). Conversely, rural households consumed roots and tubers more frequently (2.55 ± 1.61) than urban households that consumed only 1.94 times in the recall period. Further results showed significant differences in the consumption of meat/poultry/offal, milk/milk products, and sweet/sugar/honey, eggs and beverages/spices/condiments (*p* < 0.05) with the urban households overwhelming eating more of those food items compared to their rural counterparts. There were no significant differences in the consumption of fruits, vegetables, pulses, nuts, legumes, fish and seafood, oils and fats between rural and urban households.

**Table 2:** Mean frequency of food consumption between urban and rural households in Northern Uganda (*n* = 364)

Food Groups	Household location		*p*-value
	Rural	Urban	
Cereals	3.65 ± 1.56	4.12 ± 1.60	0.005***
Roots and Tubers	2.55 ± 1.61	1.94 ± 1.27	0.000***
Vegetables	2.71 ± 1.27	2.79 ± 1.12	0.512
Fruits	2.18 ± 2.10	2.13 ± 1.57	0.799
Meat, Poultry, Offal	0.50 ± 0.69	0.96 ± 0.94	0.000***
Eggs	0.20 ± 0.68	0.51 ± 0.96	0.000***
Fish and Sea food	1.89 ± 1.32	1.76 ± 1.27	0.331
Pulses, Legumes and Nuts	2.67 ± 1.18	2.80 ± 1.13	0.305
Milk and Milk Products	0.13 ± 0.44	0.45 ± 0.98	0.000***
Oil and Fats	4.07 ± 1.89	4.32 ± 1.87	0.21
Sweets, Sugar and honey	3.17 ± 2.74	4.84 ± 2.76	0.000***
Beverages /Species /Condiments	6.73 ± 0.93	6.95 ± 0.39	0.003***

Asterisks denote the level of significant difference between rural and urban locations. *** and ** denotes *p* < 0.01 and *p* < 0.05, respectively

### Household dietary diversity score

**Fig. 2:**
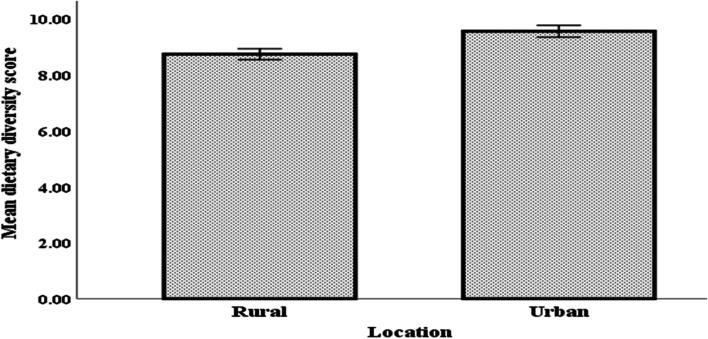
Variation in dietary diversity scores between rural and urban households based on a 7-day dietary recall period (*n* = 364)

The results of dietary diversity assessment are presented in Fig. 2 and Table 3. Figure 2 summarizes the number of food groups consumed by households on a seven-day recall period. There was a significant difference in dietary diversity score (*p* < 0.01) between rural and urban households with the latter having a higher score (9.57 ± 1.44) compared to the former (8.76 ± 1.37). In regards to grouping based on dietary diversification, Table 3 shows that majority of households, both in rural and urban locations, had high dietary diversity, having consumed at least 9 out of 12 food groups in the 7-day period preceding the survey. Overall, the dietary diversity was however, significantly higher among urban households compared to their rural counterparts by 22%.

**Table 3:** Distribution of household compliance to various dietary diversity status segregated by location of residence (*n* = 364)

Level of Dietary Diversity	Region		*P*-value
	Rural N (%)	Urban, N (%)	
Low (≤ 5 food groups)	2 (1.1)	2 (1.1)	0.000^***^
Medium (6 – 8 food groups)	71 (39.0)	31 (17.0)	
High (≥ 9 food groups)	109 (59.9)	149 (81.9)	

Asterisks denote the level of significant difference between rural and urban locations with *** denoting *p* < 0.01, ** *p* < 0.05 and* *p* < 0.10

### Status of nutritional knowledge regarding dietary diversity

**Fig. 3:**
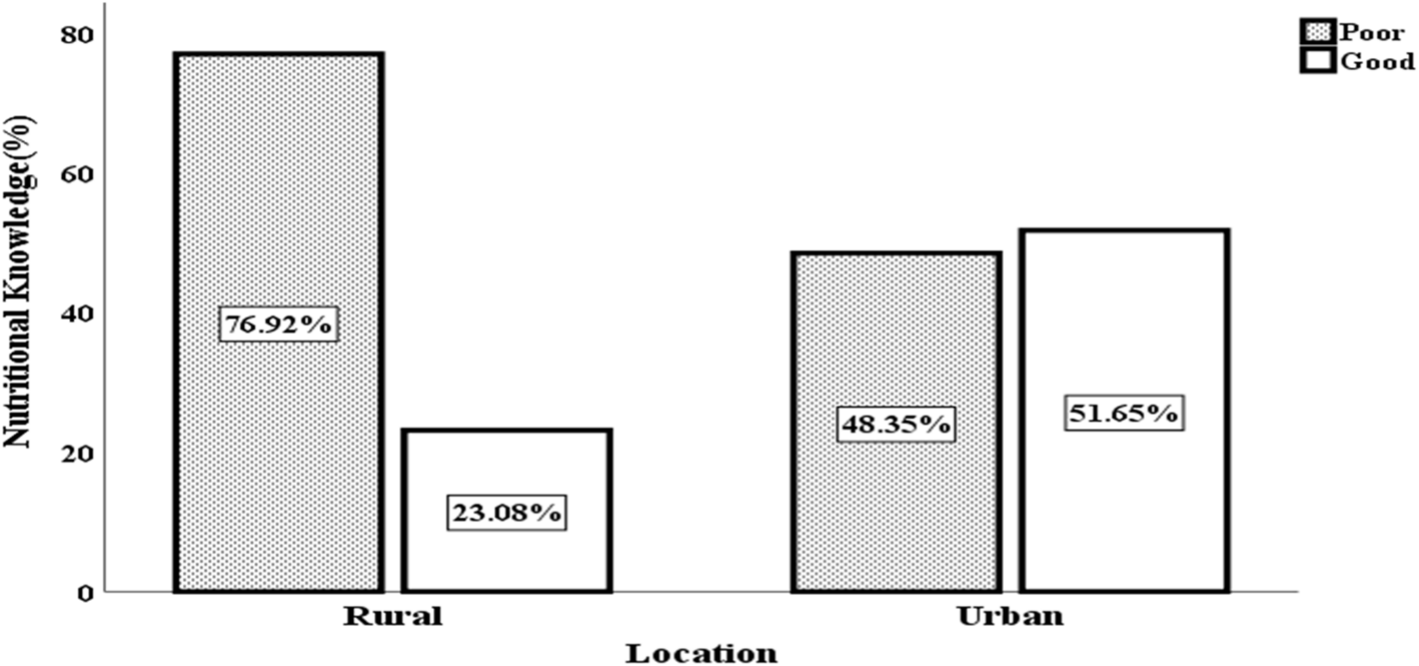
Proportion of household caregivers with poor and good level of nutritional knowledge toward dietary diversity (*n* = 364)

In Table 4, we present the proportion of households with correct nutritional knowledge toward dietary diversity. It shows that 69.8 and 81.3% of rural and urban households, respectively, were able to correctly indicate the reason why household should diversify their diets. Meanwhile, 50 and 70.3% of rural and urban household respectively were able to correctly recognize food groups that should be consumed in less quantity. About half of urban households were knowledgeable about foods that should be eaten in large quantity compared to only 26% of the rural households with such knowledge. A very low proportion of participants, both in rural and urban locations, had knowledge of energy yielding foods and food groups rich in protein. Only 21.4 and 24.7% of caregivers in rural and urban households respectively, were knowledgeable about foods with recommended fats. Overall, less than quarter of rural households had good nutritional knowledge toward dietary diversity as compared to more than half of urban households (Fig. 3).

**Table 4:** Proportion of households with correct responses to various questions on nutritional knowledge toward dietary diversity disaggregated by location of residence (*n* = 364)

Nutritional knowledge aspect tested	Rural N (%)	Urban N (%)	*p*-value
Why should household diversify diet?	127 (69.8)	148 (81.3)	0.010^**^
What are some examples of food group?	76 (41.8)	86 (47.3)	0.292
What is good nutrition?	121 (66.5)	124(68.1)	0.737
Which of these foods are energy yielding?	8(4.4)	12(6.6)	0.358
Which food groups should be consumed in less quantity?	91 (50.0)	128(70.3)	0.000^***^
Which of these foods should be consumed in large quantity?	48(26.4)	93(51.1)	0.000^***^
Which of these are protein rich foods?	6(3.3)	37(20.3)	0.000^***^
Which foods are not protein rich?	78(42.9)	90(49.5)	0.207
Which foods should not be frequently consumed?	45(24.7)	52(28.6)	0.407
Which of these are recommended fats?	39(21.4)	45(24.7)	0.455

Asterisks denote the level of significant difference between rural and urban locations with *** denoting *p* < 0.01, ** *p* < 0.05 and* *p* < 0.10

### Status of nutritional attitude regarding dietary diversity

Results in Table 5 show the proportion of households according to the status of nutritional attitude toward dietary diversity disaggregated by location.

**Table 5:** Proportion of households with various status of nutritional attitude toward dietary diversity disaggregated by location (*n* = 364)

	Nature of attitude						
Aspect of nutritional attitude tested	Negative		Neutral		Positive		*p*-value
	Rural	Urban	Rural	Urban	Rural	Urban	
Household should consume more pulses and cereals daily to maintain good health^**R**^	120(65.9)	115(63.2)	6(3.3)	9(4.9)	56(30.8)	58(31.9)	0.690
Foods rich in saturated fats should be consumed more often than unsaturated fats^**R**^	101(55.5)	78(42.9)	18(9.9)	16(8.8)	63(34.6)	88(48.4)	0.027^**^
Eating three servings of vegetables and fruits every day is very important	65(35.7)	43(23.6)	8(4.4)	8(4.4)	109(59.9)	131(72.0)	0.039^**^
Fast foods are convenient for consumption daily^**R**^	24(13.2)	21(11.5)	18(9.9)	13(7.1)	140(76.9)	148(81.3)	0.541
Slicing green vegetables before washing wash away useful nutrients	79(43.4)	49(26.9)	16(8.8)	7(3.8)	87(47.8)	126(69.2)	0.000^***^
I am convinced that spicy foods are good for all people^**R**^	87(47.8)	67(36.8)	18(9.9)	28(15.4)	77(42.3)	87(47.8)	0.068^*^
Traditional vegetables should not be cooked with other foods^**R**^	73(40.1)	59(32.4)	18(9.9)	28(15.4)	91(50.0)	95(52.4)	0.154
Eating different types of foods is always good for all people	124(68.1)	135(74.2)	19(10.4)	13(7.1)	39(21.4)	34(18.7)	0.380
I am convinced that consuming soda is always healthy^**R**^	22(12.1)	17(9.3)	11(6.0)	8(4.4)	149(81.9)	157(86.3)	0.516
Consuming eggs twice daily is good	72(39.6)	61(33.5)	17(9.3)	19(10.4)	93(51.1)	102(56.0)	0.488

Results are presented as frequencies and percentages (in bracket). Asterisks denote the level of significant difference between rural and urban locations with *** denoting *p* < 0.01, ** *p* < 0.05 and* *p* < 0.10. ^**R**^means the statement was reversed code

**Fig. 4:**
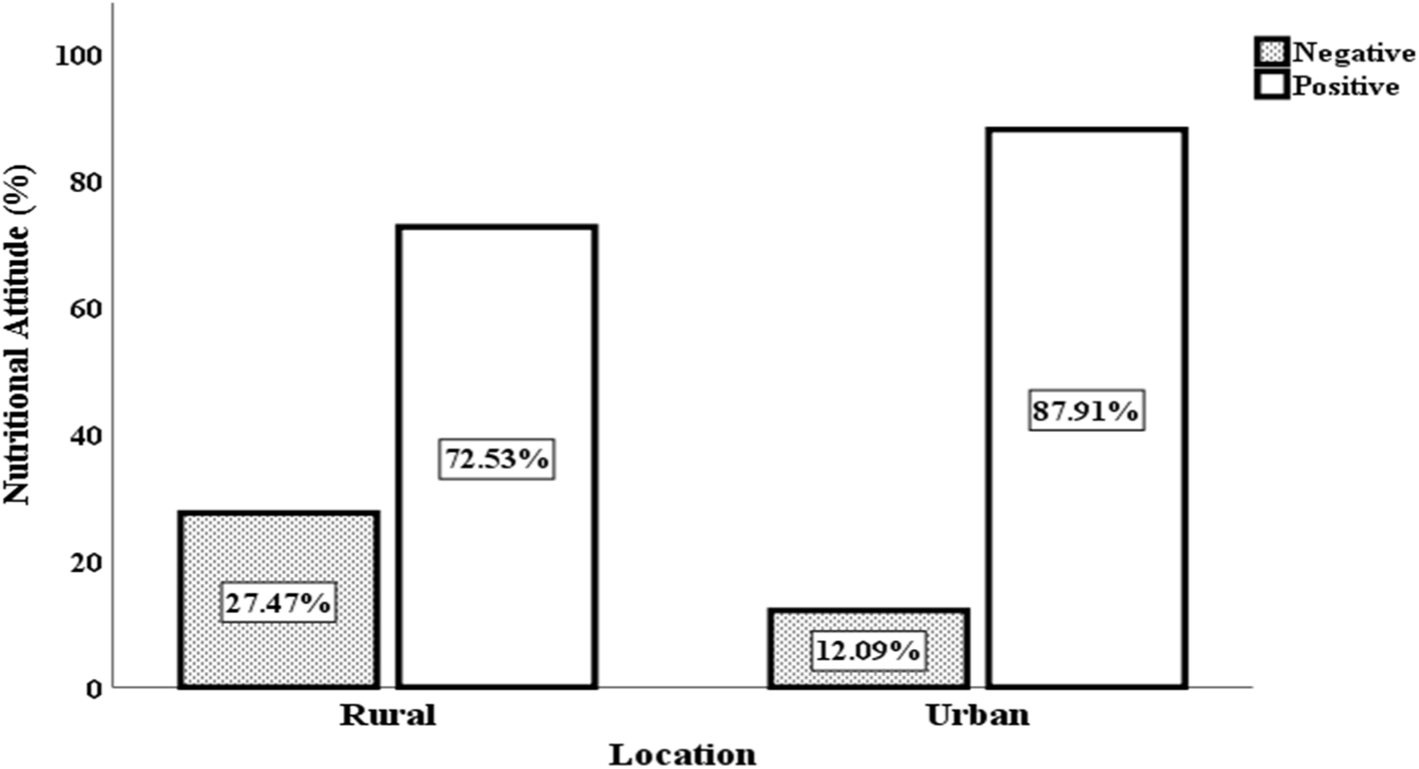
Proportion of households exhibiting negative or positive attitude toward dietary diversity disaggregated by location (*n* = 364)

The results indicate that 31.9% of urban households had a positive attitude toward vegetables and legumes when compared to rural households (30.8%). Moreover, 47.8 and 69.2% of urban and rural households respectively believed that slicing green vegetables before washing would lead to loss of nutrient. This indicate that urban households have a positive attitude toward green vegetables when compared to rural households. Besides, 86.3% of urban households had shown positive attitude toward consumption of sweets and sugar when compared to rural households (81.9%). About half of households in both locations demonstrated having positive attitude toward eggs as a food group with less than half also having positive attitude toward fats and spicy foods. On average, urban households exhibited the highest proportion of positive attitude-87.91% when compared to rural households-72.53% (Fig. 4).

### Predictors of household dietary diversity

The Poisson regression was used to estimate the predictors of dietary diversity. Results presented in Table 6 show that a unit increase in knowledge leads to an increase in the dietary diversity in the rural areas by 0.114 times compared to the urban areas. A change in attitude has no effect on household dietary diversity in rural and urban areas of Northern Uganda. Further outputs denote that in rural areas, being married is likely to decrease dietary diversity by 2.54 times when compared to urban areas in which such participants are 1.70 times more likely to diversify their diets. Factors such as education of the person in charge of food preparation, educational qualification of the household head and household food expenditure also had significant effects on household dietary diversity. Household dietary diversity would decrease by 0.840 and 0.682 in both rural and urban areas, respectively if the household caregiver had attained only primary educational qualification with the reduction being higher in the urban than rural. The same is true in the urban areas if the head of the households had attained secondary education except this change was much beneficial in the rural areas, which were more likely to diversify their diets by 0.003 times. Although household food expenditure was a good predictor of dietary diversity among rural households, a unit change in such expenditure would decrease dietary diversity by 2.96 times in that location. This is as opposed to urban households for which household food expenditure had no effect.

**Table 6:** Predictors of Household dietary diversity among rural and urban households of northern Uganda (*n* = 364)

Associated factors	Rural			Urban		
	β	δy/δx	p| >|z	β	δy/δx	p| >|z
Knowledge(Score)	0.114 (0.019)	0.990 (0.158)	0.00***	-0.008 (0.014)	-0.080 (0.135)	0.551
Attitude(1-Positive, 0-otherwise)	0.006 (0.077)	0.053 (0.667)	0.936	-0.012 (0.110)	-0.113 (1.06)	0.915
Age of Caregiver (Log)	-0.275 (0.490)	-0.044 (0.078)	0.787	-0.566 (0.508)	-0.141 (0.127)	0.265
Marital status(1-Married, 0-Otherwise)	-2.541^**^ (0.945)	-0.476 (0.180)	0.008^**^	1.700^***^ (0.630)	0.393 (0.123)	0.001^***^
Education of Caregiver (1-Primary, 0-Otherwise)	-0.840^*^ (0.438)	-0.146 (0.081)	0.073^*^	-0.682^**^ (0.324)	-0.169 (0.079)	0.032^**^
Education of Household head (1-Secondary, 0-Otherwise)	0.003^***^ (0.001)	0.001 (0.000)	0.002^***^	-0.002 (0.0006)	-0.001 (0.000)	0.011^**^
Household Size (in number)	0.518 (0.458)	0.081 (0.070)	0.248	-0.356 (0.339)	-0.088 (0.084)	0.291
Household Monthly Income ( Ugandan Shilling)	-0.235 (0.446)	-0.038 (0.074)	0.607	-0.286 (0.434)	-0.071 (0.108)	0.509
Household Food Expenditure (Ugandan Shilling)	-2.960^***^ (1.153)	-0.629 (0.143)	0.000^***^	-0.119 (0.361)	-0.030 (0.090)	0.742
Constant	5.518^**^ (2.153)			1.053 (1.774)		
Pseudo R^2^	0.175			0.061		
Prob > Chi^2^	0.000			0.031		

Asterisks denote the level of significant difference between rural and urban locations with *** denoting *p* < 0.01, ** *p* < 0.05 and *p < 0.10

## Discussions

### Status of household dietary diversity

Malnutrition is a pressing international concern for which ensuring adequate household dietary diversity is essential for mitigating the challenge and its associated consequences [39]. High level of dietary diversity is often associated with better nutritional status whereas medium or low level of diversity is strongly believed to contribute to nutrient intake inadequacy [40]. The higher level of dietary diversity score reported for urban households against the low to medium diversity levels exhibited by rural households suggests that urban inhabitants have adequate nutrient intake while those in rural areas do experience inadequacies. This observation aligns well with previous household nutritional statistics which indicate that undernutrition is more prevalent in rural than in urban areas of Northern Uganda [16].

A related study [41] reported that urban household tend to have better food purchasing power than rural households. Indeed, data on sociodemographic characteristics of the households that participated in the study show those urban households have higher income and spend more on food than rural counterpart. This could partly explain the higher dietary diversity recorded among urban than rural households. On the other hand, it should be appreciated that in a developing country setting, rural households rely largely on own food production and less on market pathway for household nutrition [42]. Thus, the observed lower dietary diversity among rural households may also be due to limited production diversity although this element was not assessed. Education is usually associated with income. This could partly explain the high dietary diversity in urban area as more than half of the urban respondents and household heads were educated which means that they have the greatest possibility of engaging in employment which serves as an indicator for better living conditions [43]. This implies that households in urban areas have more disposable income making it possible for them to purchase diverse food types. On the other hand, rural households are often challenged with non-food expenditures such as school fees, purchase of consumer durables among others. The largest share of their agricultural resources is often marketed to generate income to cover expenditure associated with non-food items [44]. This leaves them with the choice of consuming fewer food groups, which affect their dietary diversity, and promotes nutrient inadequacy. ​

Looking at dietary diversity alone without considering frequency at which various food groups are consumed may not provide a clear picture of nutrient adequacy. This is because increase in the frequency of food groups consumed can exert a multifaceted effect. It can be associated with balanced intake of essential nutrients, nutrient inadequacy or over nutrition [45]. In the current study, differentials in the frequency of consumption of various foods were observed among rural and urban households. Interestingly, the results show that rural and urban households’ diets were predominantly starch-based but of different food groups. In the case of urban households, diets were dominated by cereals such as rice, millet, maize, and sorghum that were consumed four times weekly. This is as opposed to rural diets dominated by roots and tubers such as sweet potato and cassava that were consumed three times weekly. The dominance of starch-based food groups in the diets of people in Northern Uganda is not surprising as these food commodities are highly cultivated in the region with rural inhabitants at the production frontier. Starchy tubers and cereals can easily be preserved in dry form and stored for a longer period. This could explain why these food groups dominate diets of people in the study area. Considering that starchy foods are high in energy with less amount of essential micronutrients, it is plausible to suggest that their dominance limits households from meeting the required amount of the essential nutrients including proteins and micronutrients. This is because substantial evidence exist to the effect that dominance of starchy foods in the diet is an indicator of nutrient inadequacy and by extrapolation, a factor that drives undernutrition [4, 25, 46].

It is important to note that fruits and vegetables are essential sources of micronutrients and bioactive compounds vital for human health [47]. Despite their importance, this study shows that these foods were consumed only twice a week irrespective of household location making them underutilised. These results are consistent with the outcome of a previous study conducted [18] which revealed low consumption of fruits and vegetables in Uganda. This suggests that no action has been taken to improve household consumption of fruits and vegetables thus illustrating limited use of research results to improve practice. Consumption of fruits and vegetables twice a week contravenes WHO recommendation that for a healthy living, an adult should consume at least 400 g (five servings) daily [48]**.** This implies that households in northern Uganda are not deriving the health and nutritional benefits associated with adequate intake of fruits and vegetables. Incidences of deaths associated with non-communicable diseases such as cancer, diabetes, and hypertension have reached significant levels in Uganda [49]. This is against the backdrop that adequate intake of fresh fruits and vegetables are believed to reduce the chances of developing such diseases. A concerted effort is needed to deal with this important nutritional dilemma.

Animal products including meat, eggs, fish, and milk are vital sources of protein and micronutrients due to absence of anti-nutritional factors in them. However, results show that such animal-source foods were consumed less frequently in the two areas although their consumption was slightly higher in urban compared to rural households. The low level of consumption of these products is most likely due to high cost. This is not peculiar to Gulu (northern Uganda), it has also been reported in previous studies conducted under similar contextual environment in Hoima district of Uganda [50]. However, the higher level of consumption of some of the animal products (e.g. eggs and milk) among urban compared to rural households could be due to differences in economic status. This is clearly illustrated by the sociodemographic data which show that in the urban area, household income was greater than for rural households. As expected, consumption of sugar/sweets and beverages/species/condiments were higher among urban inhabitants compared to the rural population. The underlying reason that can account for the disparity is that urban households, reportedly consumed tea and cereal related porridges that are usually prepared using sugar. The low rate of consumption of beverages and sweets among rural households can be explained by limited availability of disposable income to purchase them in addition to the fact that they are not considered essential food commodities in rural settings [51].

Consumption of legumes, pulses, and nuts was very competitive between rural and urban households. The most consumed legumes were beans and peas. More than half of the households across the two locations reported to have almost consumed beans at least three times weekly. The higher and comparative consumption of these plant protein sources among rural and urban households can be explained by the fact that these plant foods are highly produced in the area and cheap compared to animal-source protein foods. Limited intake of animal source foods indicate that legumes, pulses and nuts are consumed as alternative sources of protein in both rural and urban areas of Gulu. However, these plant foods may not deliver adequate amount of nutrients due to the presence of anti-nutritional factors (e.g. phytic, lectins, saponins) which interfere with digestibility and absorption of protein and essential micronutrients [52].

Whereas household dietary diversity score (HDDS) is a proxy measure of nutritional status which often reflects the food groups consumed, the reference period for a typical HDDS varies. Most dietary studies have utilized the 24-h recall other than the 7-day recall period as applied in the current study [53–55]. Nonetheless, a 7-day dietary diversity approach captures consumption over a longer period and as such, tends to provide a more elaborate understanding of household dietary intake in comparison to the 24-h method. This tends to also justify the high dietary diversity score observed in this study. Food consumption frequency on the other hand also measure individual and household nutrient adequacy. Both HDDS and food consumption frequency are vital for assessing nutrient intake of the population directly or indirectly. Despite the observed high HDDS for both rural and urban households reported in this study, only few food groups were more frequently consumed because each food group contributes to HDDS even if consumed only once a week. Although this tends to place both locations at the higher end of the HDDS, the low consumption of animal-based foods as well as fresh fruits and vegetables tend to show that they are nutritionally inadequate. In their study [56], indicated that although HDDS is an indicator for evaluating nutrient inadequacy, there is a need to improve the method through integration of other food evaluation techniques. This further justifies the rationale for use of HDDS and food consumption frequency measurements applied in this study.

### Status of nutrition knowledge and attitude (KA)

Adequate nutritional knowledge is a recipe for healthy living as it enhances individual and household’s choice of foods leading to consumption of a balanced meal [57]. As such, it becomes very critical for any individual or household regardless of location (rural and urban). The observed higher proportion of urban respondents with good nutritional knowledge compared to their rural counterparts could be due to better educational status of the former, compared to the latter. This is consistent with results of [58, 59] for which educational status was found to be associated with good nutritional knowledge. In the current study, more than half of urban respondents reported to have attained at least secondary level of education which could have exposed them to nutrition information. Conversely, the proportion of rural participants with at least secondary education was lower. This could have limited their exposure to nutrition knowledge. The difference in nutritional knowledge toward dietary diversity can also be attributed to the level of access to essential nutrition information disseminated through radio broadcast, electronic and print media and lesson learned from classrooms, which most rural households may not easily access. This assertion draws credence from the work of [60] which revealed that radio broadcast and classroom lessons contributed 69.4 and 69.2% of nutritional knowledge gained among school goers in Kenya. The author further showed that schools and families formed a community of practice (COP) in which nutritional knowledge was circulated. The disparity observed between rural and urban respondents is also an indication of limited attention in prioritizing rural people in nutrition programme. The limited state of nutrition knowledge in the rural area is well-illustrated by the response to the question “why a household should consume diverse food groups?” for which more than half of the respondents reported in terms of being essential for increasing household appetite for different food types.

A critical look at results reveals that rural households generally had low level of knowledge about various food groups and the types of nutrients that can be derived from them. This is because only 41.8% of households in the rural areas were able to correctly identify food groups compared to 47.3% that did the same in the urban area. This partly explains why there was general lack of basic idea on the various food recipes prepared among rural households. Given that classification of foods in groups can be learned in secondary education settings, this finding suggests that primary education which was the predominant level of education in the rural area, is not a sufficient condition for attainment of nutritional knowledge about dietary diversity.

Nutritional attitude is generally believed to be influenced by nutritional knowledge [61, 62]. However, this was not the case in the current study. In fact, nutritional attitude was generally positive compared to nutritional knowledge. The high proportion of households that exhibited positive attitude toward dietary diversity in both rural and urban areas may be explained by the abundance of different food groups during the period studied. Although nutritional attitude was positive across all locations, it was much better in the urban area. This differential suggests that certain factor(s) peculiar to urban settings might be responsible for the observed higher intensity of good attitude in the urban area. The higher level of education attainment in the urban area depicted in the sociodemographic results might be a plausible factor. This is because previous studies conducted by [10, 63] showed that good attitude toward nutrition was exhibited by 90% of the households for which respondents had attained higher level of formal education beyond primary. On the other hand, cultural norms and traditions are important factors which shape people’s attitude toward specific food types or groups [64, 65]. Previous studies reported that these practices tend to diminish in urban places because of the adoption of westernized lifestyles compared to rural areas [66, 67]. This could further explain the observed disparity in household’s attitudes toward dietary diversity between rural and urban inhabitants observed in this study.

### Predictors of dietary diversity

Several factors influence dietary diversity, key among them are education, income, land size and access to food [68]. Nonetheless, in this study, results have provided evidence on knowledge and attitude in addition to contextual background characteristics such as marital status, age, income, household food expenditure, household size and education. Nutritional knowledge is a positive predictor of dietary diversity in the rural areas compared to urban areas. While the level of knowledge was low in the rural areas, also with low dietary diversity, this result might show that the rural participants are still knowledgeable of the less complex food system (with less diverse foods) in the rural areas compared to the urban setting. On the other hand, it is perceived that people with good knowledge are more curious about what they eat and would make better food choices and therefore choose foods from diverse groups as seen with the urban consumers studied. In the urban areas, having positive attitude towards diverse diet had a significant effect on household dietary diversity. This is important in an urban setting where there are many types of food available for consumption, making attitude a key factor in choosing foods from different food groups. This observation has been made in previous related studies [10].

When factoring in the sociodemographic characteristics of the study participants, our model shows different outcomes across the two areas. Dietary diversity tended to decrease among rural households compared to urban households when the head of the household was married. Individuals entering marriage in urban households often merge their personal food systems to create a joint spousal food system [3, 69]. They tend to jointly manage household workloads including purchasing and preparation of food, which increases their knowledge about the different food groups hence promoting dietary diversity. This is rarely the case in the rural area as household food preparation decisions are often left to wives to execute. However, this differential can also be attributed to the types of food grown and consumed in rural areas. Rural households are mostly accustomed to the limited number of food they grow which tend to also limit their understanding of other food groups that exist in other settings [70].

The finding that secondary level of education of household heads had a negative effect on dietary diversity in the urban area is an interesting one. Household unitary model of decision making indicates that 90% of household food related decisions are treated singly with both couples having equal bargaining power [71, 72]. On the basis of this model, it is therefore plausible to suggest that while education is associated with employability it seems that urban spouses tend to be more engaged in their workplaces and as such, leave household food decisions to housemaid or caregiver who might not make better food decisions to effect adequate attainment of dietary diversity. In the case of rural areas, farming is usually the major source of employment. Often, food is prepared on farms for which food decisions are directly dictated and supervised by the head of the household or the spouse. Similarly, it is apparent that attainment of primary education by the household caregiver had a reduction effect on dietary diversity among both rural and urban households although the extent of this change was more significant in the urban than in the rural area. This is not surprising for the case of rural households because a person who attained primary level of education is likely to be limited in knowledge on dietary diversity. In urban areas, most household heads are heavily engaged in formal employment. Decisions toward foods prepared are left with the household caregivers, the majority of whom had attained primary education qualification. As illustrated in a study conducted by [73], most household caregivers tend to have very little understanding of the diverse food groups because primary level of education is insufficient for good nutritional knowledge.

Furthermore, it was observed that a unit change in household food expenditure in the rural area was associated with a reduction in household dietary diversity. This is likely because in the rural area, due to limited income, households would purchase fewer food groups of their preferences due to a huge constraint on household budget [42, 74, 75].This probably limits understanding of diverse food groups making households fail to meet daily nutrient requirements. This interference tends to authenticate the high rate of undernutrition reported among rural compared to urban households in Gulu in the past demographic health survey [76], generating a question as to whether rural households in Gulu district do appreciate the nutritional benefits of diverse food groups available.

## Conclusion

Urban households practice better dietary diversity than rural households in Northern Uganda. Rural households can only attain medium level of dietary diversity, which is an indication of dietary inadequacy and cannot be disassociated from increase in the rate of undernutrition. Rural and urban diets are predominantly starch-based with cereals, root, and tubers being at the consumption frontier. Urban households tend to surpass their rural counterparts in consumption of sweets and sugars and animal proteins, two distinct food groups that promote lifestyle diseases. Both rural and urban households consumed fruits and vegetables less frequently indicating that they are not meeting the WHO recommendation of 400 g daily consumption for a healthy living. Moreover, nutritional knowledge and attitude toward diverse food groups were observed to be very different across the region, with nutrition knowledge in the rural area being the worst. Although urban households had better level of nutrition knowledge, some critical aspects that define good nutrition were generally lacking. Nutritional attitude was good among both urban and rural participants although some important elements that reflect positive attitude towards dietary diversity are relatively low. In terms of the predictors of dietary diversity, nutritional knowledge, marital status, education of the caregiver, education of the household head and household food expenditure are good predictors but their effects depend on the location.

The differentials in the level of dietary diversity between rural and urban households can be harmonized through nutrition education and outreach programs. Specific efforts should be tailored toward educating rural households about the FAO recommended twelve-food groups with special attention towards consumption of fruits and vegetables.

Whereas this study has underscored the influence of nutritional knowledge and attitude, modified by socio-demographic factors on achieving recommended dietary diversity in the rural and urban settings of Northern Uganda, it had few limitations. First, it did not consider the sources of food consumed by the households, which would affect how dietary diversity is achieved and likely to vary between rural and urban households. Secondly, data were collected during the dry season (October and November), which is a period of food abundance in Northern Uganda, but the season of lean food availability (April to September) was not considered. Thus, the results are not applicable across seasons.

## Data Availability

The datasets used and/or analysed during the current study are available from the corresponding author on reasonable request.

## Abbreviations

HDDQ: Household dietary diversity questionnaire
HDDS: Household dietary diversity score
FAO: Food and Agriculture Organization of the United Nations
FFQ: Food frequency questionnaire
KA: Knowledge and attitude
